# Decision effect of a deep-learning model to assist a head computed tomography order for pediatric traumatic brain injury

**DOI:** 10.1038/s41598-022-16313-0

**Published:** 2022-07-21

**Authors:** Sejin Heo, Juhyung Ha, Weon Jung, Suyoung Yoo, Yeejun Song, Taerim Kim, Won Chul Cha

**Affiliations:** 1 Department of Emergency Medicine, Samsung Medical Center, Sungkyunkwan University School of Medicine, 81, Irwon-ro, Gangnam-gu, Seoul, 06351 Republic of Korea; 2 Department of Digital Health, Samsung Advanced Institute for Health Sciences and Technology (SAIHST), Sungkyunkwan University, Seoul, Republic of Korea; 3 Department of Computer Science, Indiana University Bloomington, Bloomington, IN USA

**Keywords:** Health care, Medical research

## Abstract

The study aims to measure the effectiveness of an AI-based traumatic intracranial hemorrhage prediction model in the decisions of emergency physicians regarding ordering head computed tomography (CT) scans. We developed a deep-learning model for predicting traumatic intracranial hemorrhages (DEEPTICH) using a national trauma registry with 1.8 million cases. For simulation, 24 cases were selected from previous emergency department cases. For each case, physicians made decisions on ordering a head CT twice: initially without the DEEPTICH assistance, and subsequently with the DEEPTICH assistance. Of the 528 responses from 22 participants, 201 initial decisions were different from the DEEPTICH recommendations. Of these 201 initial decisions, 94 were changed after DEEPTICH assistance (46.8%). For the cases in which CT was initially not ordered, 71.4% of the decisions were changed (p < 0.001), and for the cases in which CT was initially ordered, 37.2% (p < 0.001) of the decisions were changed after DEEPTICH assistance. When using DEEPTICH, 46 (11.6%) unnecessary CTs were avoided (p < 0.001) and 10 (11.4%) traumatic intracranial hemorrhages (ICHs) that would have been otherwise missed were found (p = 0.039). We found that emergency physicians were likely to accept AI based on how they perceived its safety.

## Introduction

Medical artificial intelligence (AI) modules have been developed for use in various fields^1–4^. The role of AI in medicine is quite diverse as AI is involved in a wide range of medical care processes in various ways, from diagnosis to predicting prognoses^5–9^. AI approaches can be categorized into several types based on their functions as clinical practice tools: (1) analysis of complex medical data to derive medical insights, (2) image data analysis and interpretation, and (3) monitoring continuous medical data to plan appropriate treatment strategies and follow-up^2^.

Several clinical decisions are required during routine medical care processes; accurate and rapid clinical decisions are directly linked to effective patient outcomes^10–13^. It has been proven that AI assistance in clinical decisions improves diagnoses and treatment processes in terms of accuracy and timeliness in well-designed interventions^6,14–18^. However, most studies only describe the mechanisms of AI impact using mathematical and computational methods^19^; few studies focus on the clinical decision-making process, e.g., explaining the effectiveness of a machine learning (ML)-based clinical decision support program.

Immediate clinical decision-making is particularly important in the emergency department (ED)^20–22^, where decisions such as obtaining a head CT for a pediatric patient are the most challenging^23–25^. Head CT is a simple yet critical decision, because while the head CT is a diagnostic tool of choice for intracranial hemorrhage (ICH)^26^, it is also associated with an increase in malignancies^27,28^.

Conventional head CT clinical decision rules, such as Pediatric Emergency Care Applied Research Network (PECARN) rules, have been introduced to guide clinical decisions; however, the rules require detailed history and are difficult to apply in clinical practice^29–31^. AI prediction models have been developed to overcome such limitations. Although models for head CT scans with good performance and accuracy have been developed^25,32,33^, their impact on clinical decision-making has not been explored.

This study aims to measure the effectiveness of the deep-learning model for predicting traumatic ICH (DEEPTICH) in the decisions of emergency physicians regarding head CTs, and to evaluate the factors associated with the effectiveness of DEEPTICH.

## Results

### Prediction model performance

**Figure 1:**
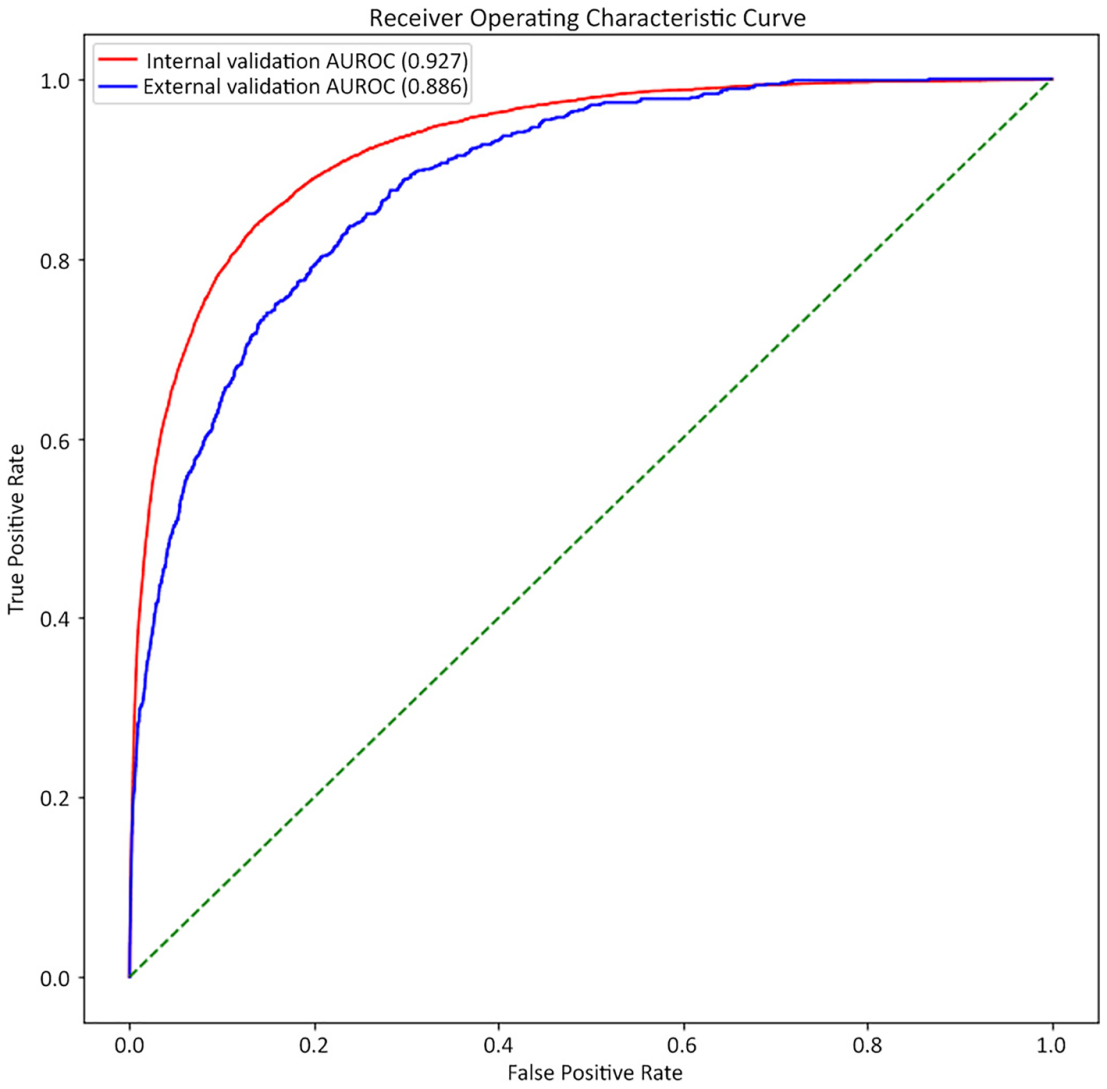
Receiver operating characteristics (ROC) curve for internal validation outcome on the time-validation set.

DEEPTICH predicted ICHs such as cerebral contusion, subdural hemorrhage, epidural hemorrhage, subarachnoid hemorrhage, intraventricular hemorrhage, intracerebral hemorrhage, and cerebellar hemorrhage, but not microhemorrhage. DEEPTICH obtained a value of 0.927 (95% CI 0.924–0.930) for the area under the receiver operating characteristic curve (AUROC) on internal validation from the 80,508 cases in the national database, and 0.886 (95% CI 0.878–0.895) AUROC on external validation based on the local hospital database (Fig. 1). In the external validation sets, the overall sensitivity, specificity, positive predictive value, and negative predictive value for clinical performance were 0.95, 0.67, 0.02, and 0.99, respectively. The specificity was lower for patients under 3 years. The detailed clinical performance values by age group are shown in Supplementary Table S1.

### Demographics for decision simulation study

A total of 22 emergency physicians completed 24 simulation test cases and surveys. We obtained 528 responses for the simulation test case. The participants comprised eight junior residents of postgraduate year (PGY) 2 and 3, nine senior residents of PGY 4 and 5, and five specialists. The most common age group was 30 to 40 years, with 12 of them (54.5%) having an average age of 31.5 years. Ten (45.5%) participants had more than five years of experience each (Table 1).

**Table 1:** Characteristics of the physician.

**Gender, n (%)**	
Male	11 (50.0)
Female	11 (50.0)
**Age, n (%)**	
20–30	8 (36.4)
30–40	12 (54.5)
≥ 40	2 (9.1)
Age, mean (SD)	31.5 (4.0)
**Physician experience, n (%)**	
Junior resident (PGY 2 & 3)	8 (36.4)
Senior resident (PGY 4 & 5)	9 (40.9)
Specialist	5 (22.7)
**Work years, n (%)**	
< 5 years	12 (54.5)
≥ 5 years	10 (45.5)

Demographic characteristics of participants (n = 22).

*PGY* post graduate year.

### The influence of recommendation directions

**Figure 2:**
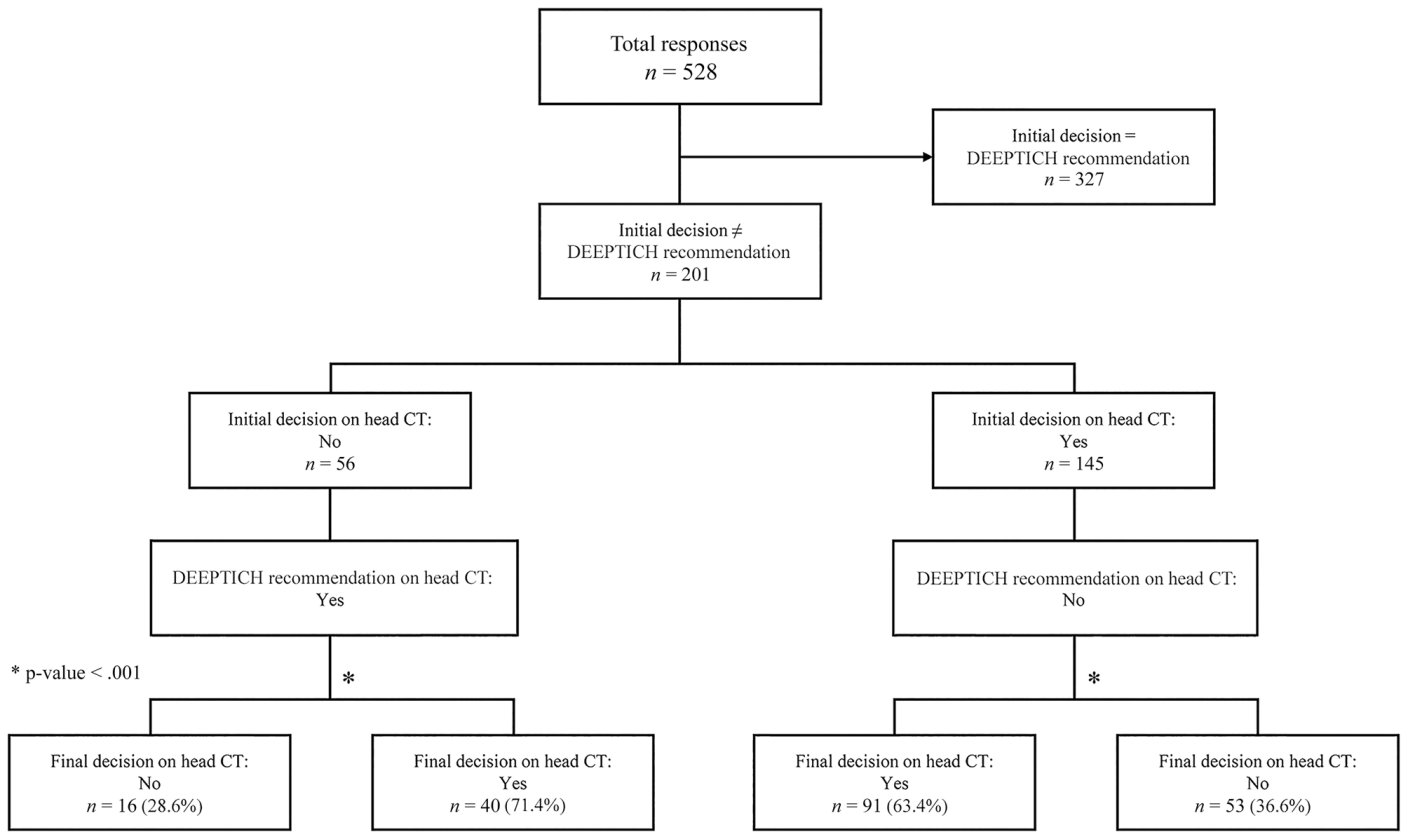
Ordering a head CT binary decision results on the simulation cases.

Figure 2 shows the flows of the responses by participants regarding the head CT binary decision before and after the DEEPTICH recommendation. The responses in which the initial decision was the same as the DEEPTICH recommendations (n = 327, 61.9%) were excluded. We analyzed the responses that were different from the initial decision and the DEEPTICH recommendations (n = 201, 38.1%).

Of the 201 responses, 56 decisions were not to order head CTs as the initial decision; however, when DEEPTICH recommended the head CT, 40 of the 56 (71.4%) decisions were changed, i.e., respondents decided to order head CTs (p < 0.001). When DEEPTICH recommended not to order head CTs, only 54 (36.6%) of the 145 initial decisions to order CTs were changed (p < 0.001).

We analyzed the responses of all the five-scale head CT ordering willingness scores based on DEEPTICH recommendation (n = 528). We found considerable score changes and decision augmentations according to DEEPTICH recommendation. In cases where DEEPTICH advised head CTs, the mean of the willingness score changed from 3.46 to 3.97 (Δwillingness, 0.51). In cases where DEEPTICH advised not to order head CTs, the mean score changed from 2.69 to 2.27 (Δwillingness, − 0.42). The detailed results are presented in Supplementary Table S2.

### The physician’s factor of influence

It was observed that when DEEPTICH recommended not to order a head CT, the decision effect differed based on the age and experience of the physician. Relatively inexperienced physicians were more likely to accept the recommendation than experienced physicians (− 43 (29%) vs. − 11 (7.3%), p < 0.001). Physicians older than 40 years did not change their decision, even though most of the physicians in the age group of 30–40 years did so: 0 (0.0%) vs. − 36 (20.0%), p = 0.021 (Table 2).

**Table 2:** Changes in the decision to order a head CT based on the physician’s characteristics.

Physician’s characteristics	DEEPTICH recommendation on head CT	Total responses	Before DEEPTICH recommendation (n, %)	After DEEPTICH recommendation (n, %)	Decision change (n, %)	p-value
**Work years**	Yes					0.689
< 5 years (n, physician number)		n = 108	78 (72.2%)	100 (92.6%)	24 (22.2%)	
≥ 5 years		n = 90	64 (71.1%)	81 (90.0%)	17 (18.9%)	
**Physician experience**						0.527
Junior resident (PGY 2 & 3)		n = 72	48 (66.7%)	64 (88.9%)	18 (25.0%)	
Senior resident (PGY 4 & 5)		n = 81	64 (79.0%)	79 (97.5%)	15 (18.5%)	
Specialist		n = 45	30 (66.7%)	38 (84.4%)	8 (17.8%)	
**Age**						0.189
20–30		n = 72	51 (70.8%)	65 (90.3%)	14 (19.4%)	
30–40		n = 108	75 (69.4%)	99 (91.7%)	26 (24.1%)	
≥ 40		n = 18	16 (88.9%)	17 (94.4%)	1 (5.6%)	
**Work years**	No					< 0.001
< 5 years		n = 180	83 (46.1%)	40 (22.2%)	− 43 (23.9%)	
≥ 5 years		n = 150	62 (41.3%)	51 (34.0%)	− 11 (7.3%)	
**Physician experience**						0.073
Junior resident (PGY 2 & 3)		n = 120	51 (42.5%)	25 (20.8%)	− 26 (21.7%)	
Senior resident (PGY 4 & 5)		n = 135	57 (42.2%)	36 (26.7%)	− 21 (15.6%)	
Specialist		n = 75	37 (49.3%)	30 (40.0%)	− 7 (9.3%)	
**Age**						0.021
20–30		n = 120	50 (41.7%)	32 (26.7%)	− 18 (15.0%)	
30–40		n = 180	76 (42.2%)	40 (22.2%)	− 36 (20.0%)	
≥ 40		n = 30	19 (63.3%)	19 (63.3%)	0 (0.0%)	

n (%) of physicians who answered ordering a head CT.

Post hoc analysis. 30–40 vs ≥ 40, p < 0.001.

### Factors associated with AI acceptance

We conducted univariate and multivariate logistic analyses to identify the factors associated with the effectiveness of DEEPTICH. The participants were more likely to accept AI recommendations when the PECARN risk was high (Odds ratio (OR), 15.02; 95% CI 1.60–473.38) and when the initial head CT decision was no (OR, 2.68; CI 1.08–6.88) (Table 3). We also conducted a logistic regression on the survey outcomes; no item was associated with the effectiveness of DEEPTICH.

**Table 3:** Logistic regression analysis of factors affecting effectiveness of machine learning model to assist head CT order.

	Univariate			Multivariate		
	OR	95% CI	p*-*values	OR	95% CI	p-values
**Gender**			0.962			
Female	(Reference)					
Male	1.01	(0.58–1.77)	0.962			
**Age**			< 0.001			
20–30	(Reference)					
30–40	1.55	(0.85–2.84)	0.154	2.10	(0.88–5.18)	0.100
≥ 40	0.06	(0.00–0.32)	0.008	0.09	(0.00–0.86)	0.078
**Physician experience**			0.006			
Junior resident (PGY 2 & 3)	(Reference)					
Senior resident (PGY 4 & 5)	0.71	(0.37–1.34)	0.289	0.87	(0.31–2.44)	0.795
Specialist	0.30	(0.14–0.63)	0.002	0.89	(0.15–5.21)	0.896
**Work years**			< 0.001			
< 5 years	(Reference)					
≥ 5 years	0.33	(0.18–0.59)	< 0.001	0.4	(0.12–1.27)	0.124
**PECARN risk**			< 0.001			
Low	(Reference)					
Intermediate	1.62	(0.90–2.93)	0.104	0.93	(0.43–2.01)	0.859
High	25.91	(4.91–479.14)	0.002	15.02	(1.60–473.88)	0.045
**Risk—age**			0.014			
< 2	(Reference)					
≥ 2	0.49	(0.28–0.87)	0.014	0.66	(0.34–1.29)	0.222
**Initial CT binary decision**			< 0.001			
Yes	(Reference)					
No	4.21	(2.19–8.42)	< 0.001	2.68	(1.08–6.88)	0.036

*PGY* post graduate year.

### DEEPTICH effectiveness by PECARN risk

We analyzed head CT decisions prior to and after DEEPTICH recommendation using PECARN risk rules (Table 4). For cases of age less than 2 years old, using DEEPTICH could have avoided 15 (13.6%) unnecessary CTs (p < 0.001) for every 110 low risk head injury patients. For every 44 high risk head injury patients, 15 (34.1%) necessary CTs were properly ordered (p < 0.001). For cases older than 2 years old, using DEEPTICH could have avoided 18 (16.4%) unnecessary CTs (p < 0.001) for every 110 low risk head injury patients. DEEPTICH did not induce significant decision changes in the intermediate group for all ages.

**Table 4:** Ordering a head CT decision result according to risk of cases.

Age	PECARN risk	Before DEEPTICH recommendation, n (%)	After DEEPTICH recommendation, n (%)	p-value
< 2 years	Low (n = 110)	37 (33.6)	22 (20.0)	< 0.001
	Intermediate (n = 110)	49 (44.5)	54 (49.1)	0.372
	High (n = 44)	28 (63.6)	43 (97.7)	< 0.001
≥ 2 years	Low (n = 110)	53 (48.2)	35 (31.8)	< 0.001
	Intermediate (n = 110)	76 (69.1)	74 (67.3)	0.619
	High (n = 44)	44 (100.0)	44 (100.0)	–

Table 5 presents the overall clinical outcomes using DEEPTICH. When using DEEPTICH, 46 (11.6%) unnecessary CTs were avoided (p < 0.001) and 10 (11.4%) all traumatic ICHs that would have been otherwise missed were found (p = 0.039).

**Table 5:** Clinical impact of the model in the study.

Case characteristics	Before DEEPTICH recommendation, n (%)	After DEEPTICH recommendation, n (%)	p-value
Avoidable head CT cases^a^ (n = 396)	191 (48.2)	145 (36.6)	< 0.001
Any ICH findings in head CT (n = 88)	70 (79.5)	80 (90.9)	0.039

*ICH* intracranial hemorrhage, *n, (%)* the number of responses who answered ordering a head CT.

^a^Avoidable head CT cases were defined as those whose CT results were finally negative in a low and intermediate risk.

### Survey outcome

The survey outcomes are presented in Table 6. Regarding general medical AI, only seven (31.8%) participants had prior experience, and five (22.7%) had the technical knowledge. In addition, 16 participants (72.7%) did not have sufficient knowledge regarding data-driven AI, but had the intention to learn, and their belief in the positive impacts of AI was significantly high (Intent, 20 [90.9%]; Optimism, 21 [95.5%]).

**Table 6:** Participant’s response to survey (n = 22).

	Agree n (%)	Disagree n (%)
**AI in general**		
Prior experience Did you attend the lecture or seminar regarding medical AI?	7 (31.8)	15 (68.2)
Technical knowledge Have you learned about coding such as C language for tensorflow, python, and R?	5 (22.7)	17 (77.3)
Knowledge Do you have enough knowledge of data driven AI?	6 (27.3)	16 (72.7)
Optimism Do you think AI will have a positive impact on medicine?	21 (95.5)	1 (4.5)
Intent Do you have an intention to learn medical AI?	20 (90.9)	2 (9.1)
**AI in this study (DEEPTICH)**		
Comprehension I understood the mechanism of the DEEPTICH	19 (86.4)	3 (13.6)
Reliability I trust the recommendation of the DEEPTICH	19 (86.4)	3 (13.6)
Clinical safety I believe that DEEPTICH is safe for use in clinical settings	15 (68.2)	7 (31.8)
Perceived compatibility The recommendation of the DEEPTICH is easy to understand	20 (90.9)	2 (9.1)
Information quality The quality of DEEPTICH recommendation is sufficient to make a clinical decision	17 (77.3)	5 (22.7)
Doctor-patient relationship DEEPTICH can help improve doctor-patient relationships	18 (81.8)	4 (18.2)
Potential effectiveness DEEPTICH can be clinically effective in improving patient treatment quality and prognosis	19 (86.4)	3 (13.6)

Among the participants, 19 (86.4%) responded that they understood the mechanisms of DEEPTICH. The participants mostly disagreed with DEEPTICH regarding clinical safety. Only 15 (68.2%) participants agreed with the recommendations on clinical safety, whereas five (22.7%) participants disagreed with the quality of information obtained from DEEPTICH.

## Discussion

To the best of our knowledge, this is the first study to develop a decision simulation study design and to investigate the acceptance of AI in clinical decisions by physicians. Most AI-based clinical decision support system (AI-CDSS) studies have reported improved diagnostic accuracy or efficiency based on the agreements with AI by doctors^34,35^. However, before considering the effectiveness of an AI approach on accuracy, it is necessary to know in detail its function in the clinical decision-making process.

In this study, we developed DEEPTICH, a deep-learning model for predicting traumatic ICHs. DEEPTICH had higher AUROC than previously known pediatric head CT rules^36^. Because the rate of traumatic brain injury (TBI) is higher in younger age groups than in older age groups, this difference in data was considered when setting the model threshold, which made DEEPTICH have less specificity in younger age group^37,38^.

Subsequently, we identified that the effect of AI on decision making of the physician is influenced by various factors; one of those factors is the recommendation direction (positive vs. negative). We found that when the suggestion direction of the model is positive, emergency physicians are more likely to accept the recommendations of the model; whereas, when the suggestion direction of the model is negative, the decision change differs based on work years and age of the physician, suggesting that inexperienced clinicians are significantly more likely to be influenced by AI tools than experienced clinicians.

We demonstrated that DEEPTICH is effective, even when the AI-CDSS and the initial decisions of physicians are the same. After realizing that AI-CDSS concur with their initial decision, the level of confidence increased significantly, which is important, because clinical decisions are often challenged by non-clinical factors, both socially and psychologically.

As DEEPTICH only predicts ICHs, excluding microhemorrhages, there may be some reluctance in adopting its recommendations by clinicians because microhemorrhages are a clinically important sign of significant diffuse TBI. Despite ICH being the most common pediatric TBI for neurosurgical intervention^39,40^, for a more effective and reliable model, the prediction of other abnormalities must be considered.

We believe that DEEPTICH can make an impact in improving clinical outcomes. Overall, DEEPTICH is helpful in reducing unnecessary head CTs and missed ICH cases. Although, the model decision effect is not significant in the intermediate group, approximately 70% of children in the low- or high-risk groups of head trauma can benefit from using DEEPTICH through enhanced ordering head CT in high risk groups and decreasing ordering head CT in low risk groups.

The survey outcome indicated that physicians were concerned about the clinical safety and information quality of DEEPTICH. Therefore, we propose that for the clinical use of medical AI, development information, such as data processing and modeling should be described in a greater detail to physicians to alleviate their concerns.

Consequently, considering the results and sensitivity of DEEPTICH, we suggest using DEEPTICH with conventional head CT rules in optimizing the prevention of adverse outcomes and unnecessary head CTs. This model can be used to supplement standard head CT rules even if the case history is not filled out or it has been 24 h since the last visit, especially for doctors with less experience.

This study has two limitations. First, the simulation cases are not representative of real-world pediatric TBI populations; there is a greater proportion of low-risk TBI patients in the real world, therefore the decision effect and clinical performance of the model may be different. Second, the simulation cases were non-randomly selected; in the selected cases, DEEPTICH results were correlated to real cases. Therefore, we did not evaluate the accuracy and superiority of DEEPTICH in this well-designed decision simulation study. When implementing DEEPTICH in a real-world clinical setting, the rate of AI acceptance by the physician might be different.

We found that AI acceptance was affected by multiple factors, such as the characteristics of the physician, risk of cases, and the recommendation of DEEPTICH, making it difficult to predict the effect of the model in the real world. Therefore, when implementing AI CDSS in clinical scenarios, we suggest considering the model performance along with its acceptance by physicians. To assess improvements in the clinical outcomes, randomized clinical trials in real-world setting are required.

## Conclusions

DEEPTICH affects decisions of emergency physicians to order head CTs, as demonstrated by the decision simulation study. The effectiveness of the model is more significant when the model recommends ordering of head CTs.

## Methods

This study was approved by the Institutional Review Board (IRB) of Samsung Medical Center IRB Nos. 2020-07-072 and 2020-09-218. We conducted the decision simulation study from April 26, 2021 to June 5, 2021. Informed consent was obtained from all participants. We confirm that all the experiments were performed in accordance with the relevant guidelines and regulations.

### Deep-learning model for predicting traumatic intracranial hemorrhages (DEEPTICH) development in clinical decision support system (CDSS)

#### Dataset for deep learning

Two data sources were used in this study: the ED-based injury in-depth surveillance (EDIIS) database, and the trauma registry database of the Samsung Medical Center (SMC). The EDIIS dataset was used for model training and internal validation; the SMC dataset was used for external validation and to investigate the effectiveness of DEEPTICH. Detailed data selection criteria are provided in Supplementary Fig. S1.

The EDIIS database was established based on the International Classification of External Causes of Injuries by the World Health Organization. The database includes prehospital records, clinical findings, diagnosis, treatment, dispositions in the ED, inpatient information, demographics, and injury-related factors of the patients. Information of 1.8 million patients from 25 EDs were included in this surveillance database. Each participating hospital assigned coordinators for data collection and management, and the Korea Centers for Disease Control regularly checked the quality of the entire data from the 25 EDs. In this study, the records of 750,000 patients with head injuries from January 1, 2011 to December 31, 2017 were used for derivation and time-split validation.

The SMC database contains medical records from a tertiary academic hospital in South Korea with approximately 2000 beds and an average of 200 ED patient visits per day. This database includes the records of procedures and clinical notes, as well as the same information collected in EDIIS. The SMC dataset was collected from January 1, 2012 to December 31, 2019, with 67,578 patient records. These data were used for multi-center validation in this study.

#### Model training for deep learning

We used the patient demographics, vital signs, mental status, injury-related factors, date and time-related information regarding the injury onset and visit, and symptoms for predictors. Demographics included the age and sex. Vital signs included the respiratory rate, body temperature, systolic and diastolic blood pressure, and pulse rate. Mental status data included the “alert, verbal, pain, unresponsive” scale and Glasgow coma scale (GCS) scores. Injury-related factors included the injury mechanism, activity during the injury, alcohol-related factors, intentions, place of the injury, material causing the injury, and the time taken from injury onset to the visit. Time-related predictors included the injury onset and visit date and time information, such as the day of the week and hour of the injury onset.

Multiple outcomes were used for training the model. The primary outcome was ICH, such as cerebral contusion, subdural hemorrhage, epidural hemorrhage, subarachnoid hemorrhage, intraventricular hemorrhage, intracerebral hemorrhage, and cerebellar hemorrhage. Other outcomes, such as TBIs other than ICH, visit dispositions, and operations related to head injuries were considered as secondary outcomes. The purpose of secondary outcomes was to improve the prediction performance of the model in multi-task learning.

#### Machine learning algorithm

Multi-task learning was used for the ML algorithm to classify the ICHs and secondary outcomes. Multi-task deep learning is a method of training multiple learning tasks simultaneously during the training phase. The advantage of multi-task learning is that it can exploit useful information based on the commonalities and differences in the different tasks during training. In our case, there were commonalities and differences in hemorrhages and other TBIs, visit dispositions (patients with ICHs and serious TBIs are more likely to be admitted to the hospital), and head-injury related operations (some ICHs require acute interventions).

#### Algorithm threshold selection

There were numerous options to select an appropriate threshold for binary prediction for the DEEPTICH, including the Youden index, thresholds for generating the best F-1 score, 0.97/0.95/0.9 sensitivity, and mean threshold among groups of populations whose outcome was 1. We generated case examples for each option, and each option was reviewed by a clinician. Finally, the best threshold selected as a negative predicted value in each age group was 0.99 because it best reflected the clinical decision-making in real clinical circumstances.

### Participants for decision simulation study

The participants were residents and specialists in ED of the SMC, a single tertiary academic hospital in South Korea. We defined DEEPTICH effectiveness as a change in a head CT decision based on the DEEPTICH recommendation when the initial decision of the participant differed from the DEEPTICH recommendation. We calculated the DEEPTICH effectiveness mean and SD for five emergency physicians, which were respectively 51.0% and 10.6%. We derived the appropriate number of participants as 20, with the width of the 95% confidence interval as ± 5%. We conducted a study involving 22 emergency physicians, while considering study failures.

### Simulation cases selection

We conducted a simulation study using pediatric cases because the decision of ordering a head CT in the pediatric population is more challenging than that in adult patients. We selected 24 simulated pediatric cases who visited the ED with a fall down mechanism from the SMC validation dataset. We stratified cases based on the PECARN rule and patient age (Supplementary Table S3).

This study focused on the effectiveness of AI regarding the decision of the physician, not its accuracy, and therefore selected only cases in which DEEPTICH had the same results as the patient outcome. We performed a trend test (Cochran Armitage test) and Spearman correlation analysis to verify the validity of the simulated cases. For high-risk cases, we confirmed that the extent of the decision on the head CT ordering was significant, and that the head CT ordering willingness on a five-point scale was high.

Four sub-questions were asked for each case. Sub-questions 1 and 2 were asked before the DEEPTICH recommendation, and sub-questions 3 and 4 were asked after the DEEPTICH recommendation. Sub-questions 1 and 3 were identical to the head CT order binary decision. Sub-questions 2 and 4 were also identical to those concerning the willingness of head CT ordering, i.e., five-point scale score. The participants answered all four sub-questions. DEEPTICH presented three pieces of information: (1) the ICH probability of the case; (2) a top percentage of probability of an ICH in the same age group; and (3) a head CT order decision from the DEEPTICH. The process of the simulation scenario is shown in Textbox 1.

**Textbox 1:**
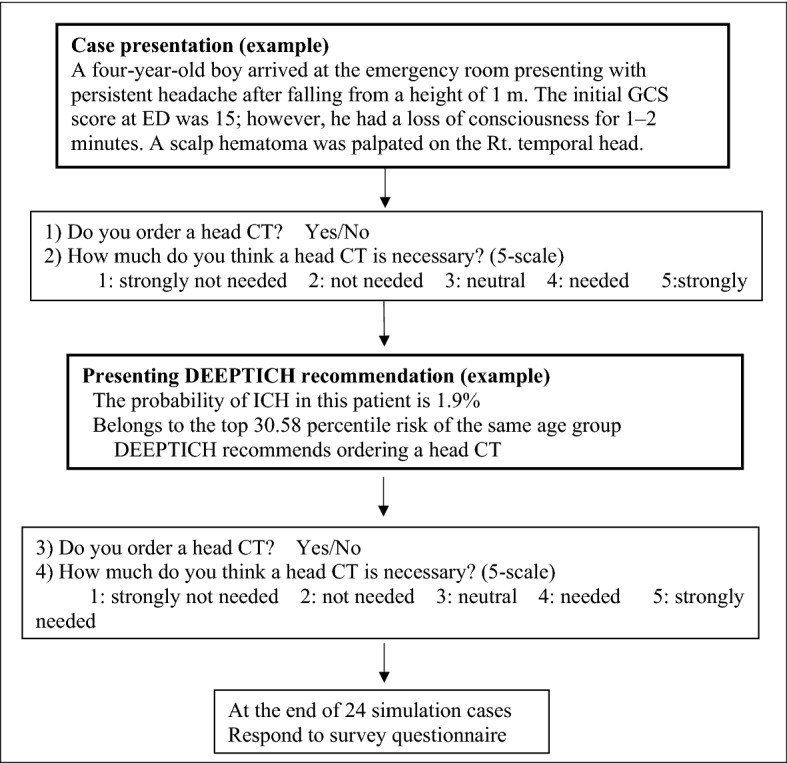
Process of simulation scenario.

### Survey development

We performed a survey to investigate the factors affecting the DEEPTICH effectiveness. The survey consisted of five questions regarding general medical AI and seven questions regarding the AI used in this study (i.e., the DEEPTICH).

### Study process

Consent was obtained from all participants. The participants were provided with the model information and the PECARN rules. Using the PECARN rules was left to the discretion of the physician, and its frequency was not measured. We explained the characteristics and development of DEEPTICH and its clinical performance, i.e., the sensitivity, specificity, negative predictive value, and positive predictive value. Details of model development are in the Supplementary Method. The participants were asked to answer four sub-questions for each simulation case: they were required to answer two questions before the DEEPTICH recommendation and the same two questions after DEEPTICH recommendation (Textbox 1). The simulation case was viewed in a Q-card format. After the completing the 24 simulation cases, the participants responded to the survey.

#### Outcomes

The primary outcome was the change in head CT order binary decision when the initial binary decision was different from the DEEPTICH recommendation. The secondary outcome was the change in the five-point willingness scale score, and factors that affect the decision changes. We determined that DEEPTICH was effective when the physicians changed their binary decisions based on the DEEPTICH recommendation.

### Statistical method

We used the McNemar test for the changes in the proportion for the paired data, and the paired t-test for the continuous values to conduct comparisons before and after the DEEPTICH recommendation. We conducted univariable and multivariable logistic regression analyses to evaluate the factors associated with the DEEPTICH effectiveness. p < 0.05 was considered as statistically significant for all statistical tests. The R software (Version 4.0.2) was used for the statistical analysis.

## Supplementary Information

Supplementary Information 1.

Supplementary Information 2.

## Data Availability

Due to Korea Centers for Disease Control and Prevention regulations, the raw data for deep learning are not publicly available. Upon reasonable request, the corresponding author can provide simulation examples and data that support the findings of this study.
