# Supplementary material for: Decision effect of a deep-learning model to assist a head computed tomography order for pediatric traumatic brain injury
Source: Sci Rep. 2022 Jul 21;12:12454. doi: 10.1038/s41598-022-16313-0 (PMC9304372; doi:10.1038/s41598-022-16313-0)
Supplement: Supplementary file 1 — Supplementary Information 1. [file 41598_2022_16313_MOESM1_ESM.docx]

**Supplementary Figure S1.** Patient selection diagram for two different data sources (a) EDIIS and (b) SMC.


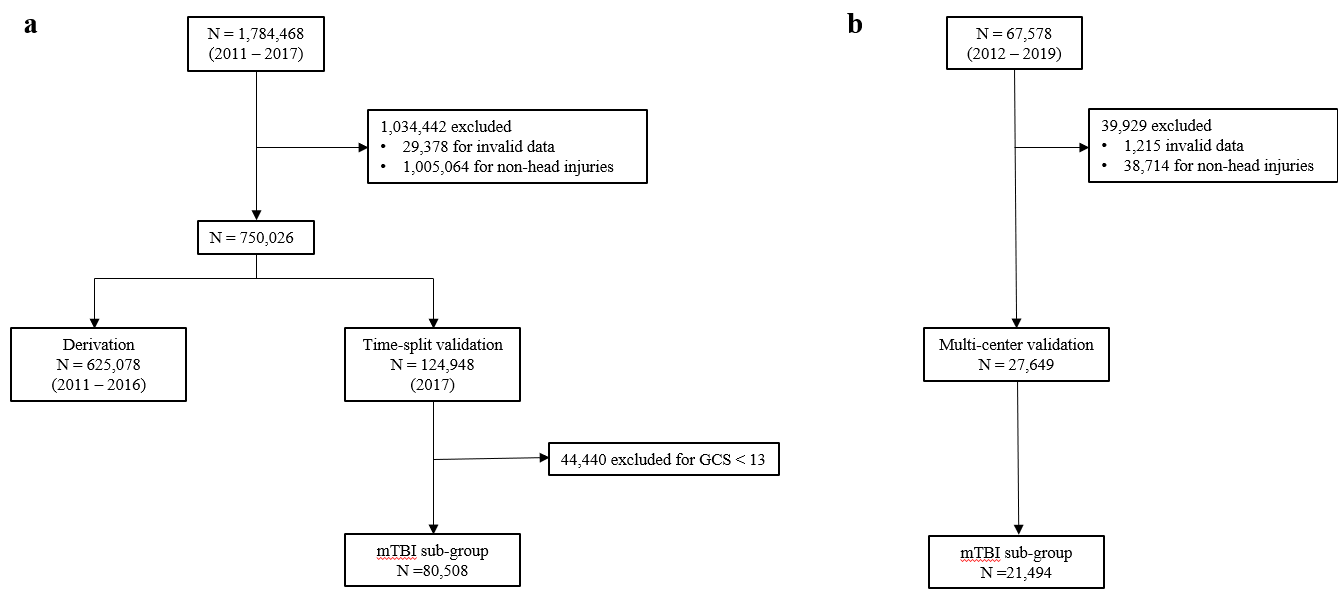
Abbreviations: *EDIIS* the emergency department-based injury in-depth surveillance, *SMC* Samsung medical center, *mTBI* mild traumatic brain injury.

**Supplementary Table S1.** Model clinical performance by age group

|  | **Age group** | **Negative predicted value (95% CI)** | **Positive predicted value (95% CI)** | **Sensitivity**  **(95% CI)** | **Specificity**  **(95% CI)** |
| --- | --- | --- | --- | --- | --- |
| DEEPTICH | 0≤ X < 1 | 0.990 (0.993-1.000) | 0.027 (0.019-0.038) | 0.865 (0.715-0.876) | 0.441 (0.426-0.457) |
|  | 1≤ X < 3 | 0.990 (0.995-1.000) | 0.030 (0.020-0.045) | 0.977 (0.832-0.992) | 0.574 (0.567-0.582) |
|  | 3≤ X < 6 | 0.990 (0.981-1.000) | 0.026 (0.024-0.028) | 0.955 (0.830-1.000) | 0.700 (0.698-0.709) |
|  | 6≤ X < 12 | 0.990 (0.986-0.995) | 0.014 (0.011-0.035) | 0.866 (0.758-0.974) | 0.714 (0.705-0.724) |
|  | 12≤ X <18 | 0.990 (0.983-0.997) | 0.023 (0.017-0.029) | 0.936 (0.850-1.000) | 0.703 (0.689-0.717) |
|  | Overall | 0.990 (0.983-1.000) | 0.018 (0.016-0.020) | 0.950 (0.912-0.988) | 0.672 (0.669-0.675) |

**Supplementary Table S2.** Five scale head CT ordering tendency score results on the simulation cases

| **Initial decision on head CT** | **DEEPTICH recommendation on head CT** | **Before DEEPTICH recommendation Mean (SD)** | **After DEEPTICH recommendation Mean (SD)** | **Tendency (SD)** |
| --- | --- | --- | --- | --- |
| **Overall**  **(**n = **528)** | Yes (n = 198) | 3.46 (1.13) | 3.97 (0.80) | 0.51 (0.78) |
|  | No (n = 330) | 2.69 (0.99) | 2.27 (0.83) | -0.42 (0.62) |
| **Yes**  **(**n = **287)** | Yes (n = 142) | 3.98 (0.87) | 4.23 (0.71) | 0.24 (0.61) |
|  | No (n = 145) | 3.57 (0.74) | 2.82 (0.83) | -0.75(0.66) |
| **No**  **(**n = **241)** | Yes (n = 56) | 2.14 (0.44) | 3.33 (0.67) | 1.20 (0.75) |
|  | No (n = 185) | 1.99 (0.47) | 1.83 (0.51) | -0.16 (0.44) |

**Supplementary Table S3.** Simulation cases matrix (n = 24)

|  | **PECARN risk** | | |
| --- | --- | --- | --- |
| Age | Low (n = 10) | Intermediate (n = 10) | High (n = 4) |
| ˂ 2year-old (n = 12) | 5 | 5 | 2 |
| ≥ 2year-old (n = 12) | 5 | 5 | 2 |

**Supplementary Table S3.** Ordering a head CT decision result according to the risk of cases

| Age | PECARN risk | Before DEEPTICH recommendation, n (%) | After DEEPTICH recommendation, n (%) | *P*-value |
| --- | --- | --- | --- | --- |
|  |  |  |  |  |
| < 2 years | Low (n = 110) | 37 (33.6) | 22 (20.0) | <0.001 |
|  | Intermediate (n = 110) | 49 (44.5) | 54 (49.1) | 0.372 |
|  | High (n = 44) | 28 (63.6) | 43 (97.7) | <0.001 |
| ≥ 2 years | Low (n = 110) | 53 (48.2) | 35 (31.8) | <0.001 |
|  | Intermediate (n = 110) | 76 (69.1) | 74 (67.3) | 0.619 |
|  | High (n = 44) | 44 (100.0) | 44 (100.0) | - |
