# Supplementary material for: Decision effect of a deep-learning model to assist a head computed tomography order for pediatric traumatic brain injury
Source: Sci Rep. 2022 Jul 21;12:12454. doi: 10.1038/s41598-022-16313-0 (PMC9304372; doi:10.1038/s41598-022-16313-0)
Supplement: Supplementary file 2 — Supplementary Information 2. [file 41598_2022_16313_MOESM2_ESM.docx]

Information about the DEEPTICH development and validation process

1. Materials and Methods

**1.1 Study cohort**

There were two data sources used in this study: the ED-based injury in-depth surveillance (EDIIS) data, and the trauma registry database of the Samsung Medical Center (SMC). From EDIIS, we chronologically divided the records into derivation (625,078 records from 2011 to 2016) and time-split validation (124,948 records from 2017). There were 318,016 (42.40%) pediatric cases in the derivation set and 32,519 (40.39%) pediatric cases in the time-split validation set. Multi-center validation used all records from the SMC database (27,649 records from 2012 to 2019). In the SMC database, there were 11,446 pediatric cases out of 27,649 (41.40 %).

In addition, we extracted a subgroup of patients with mild Traumatic Brain Injury (mTBI). This group of patients had a Glasgow Coma Scale (GCS) score greater than 13, making it difficult for clinicians to evaluate whether head CT is necessary. 80,508 patient records from the time-split validation dataset (EDIIS) and 21,494 patient records from the multi-center validation dataset (SMC) were selected for this subgroup.

**1.2 Predictors and outcomes**

We used a total 27 predictors for model development. The detailed variables are described in manuscript p.11 line 239-248. Multiple outcomes were used for the training model. The primary outcome was intracranial hemorrhage (ICH) including cerebral contusion, subdural hemorrhage, epidural hemorrhage, subarachnoid hemorrhage, intraventricular hemorrhage, intracerebral hemorrhage, and cerebellar hemorrhage. Secondary outcomes were operation, other TBIs than ICH, and visit disposition. Furthermore, there are auxiliary outcomes such as cerebral concussion and accompanying other diagnosis. We set an outcome based on ICD-10-CM codes. For classification tasks in deep learning, researchers sometimes add auxiliary output layers with variables that have some correlation with the main target outputs to improve classification performance. The reason for adding these auxiliary outcomes is that we assume there are correlations between the types of head injuries and our main outcomes and multitask learning can use this information during training.

**1.3 Data processing**

There are two major types of variables, numerical and categorical. For the numerical variables, we first imputed -1 for outliers and missing values. We did not use any scaling, such as normalization. The numerical variables included age, overall GCS score, respiratory rate, diastolic and systolic blood pressures, pulse rate, body temperature, and the time taken from injury onset to ED visit. For categorical variables, there are different types of variables, including binary, multi- class, multi-label, and text. The binary variables are as 0s and 1s. The multi-class type represents categorical data that can have only one value out of multiple candidates. For example, a patient can have only one visit disposition out of multiple values, such as discharge, hospital admission, intensive care unit (ICU) admission, and death. The multi-label type represents a list of values from multiple candidates. For example, a patient can have multiple diagnosis codes out of all possible diagnoses. Finally, the symptoms were recorded in the text. Each patient had zero to multiple symptoms, and each symptom had one to nine words.

**1.4 Model development**

We performed multitask learning (MTL) based on deep learning for our prediction model. As regards model architecture, our model comprises two input layers for symptoms (5,963 dimensions) and other input features (1,219 dimensions), including numerical, categorical, and multi-class variables (Figure 1). The input layer for symptoms is first fed to the embedding layers (100 dimensions) and then concatenated to the other input layers as a concatenating layer (1,319 dimensions). After the concatenating layer, there are eight hidden layers (each 512 dimensions) with dropout (with a 20% dropout rate) and batch normalization layers, and eight output layers (one for primary and three secondary outcomes and two for auxiliary outcomes). The outcome layers consist of three binary output layers (each having 1) for ICH, other TBI diagnosis than ICH, and operation, one multi-class output layer (four dimensions) for visit disposition.

For hyper-parameter searching and choosing the best model, the model snapshots were taken in every epoch during training, and the snapshot that performed best on the primary outcome was selected as the final model. The software implemented for model development were Python programming language (version 3.8.5), TensorFlow framework (version 2.3.1), and scikit-learn (version 0.23.2).

**Figure 1. Model architecture**


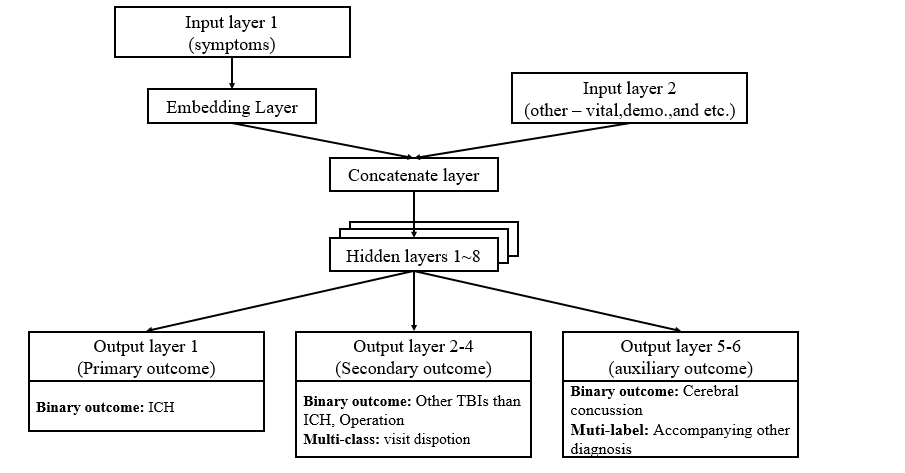


2. Results

**2.1 Patient characteristics**

Overall 750,026 patients’ basic characteristics showed that there were predominantly more males (65.4%) suffering from head trauma and their average age was 29.3 (standard deviation, 24.6); most patients had an acute mental status with a GCS score of 14.8, on average (standard deviation, 1.2). In terms of outcome distribution, 5.0% of patients experienced ICH and 1.3% of patients underwent surgery. And most patients (90.4%) were discharged without admission; 6.1% were admitted to the general ward, and only 3.5% were admitted to the ICU or died. As regarding the pediatric populations, 318,016 (42.40%) was under 18 years old and in mTBI sub-groups, there were 32,519 (40.39%) pediatric patients. In mTBI sub-groups, only 3,605 (1.13%) had ICH and 938 (0.29%) underwent operation. The injury-related characteristic showed that a large portion (37.9%) of patients were injured in their homes, 73.5% of patients visited ED by walk-in, 39.1% patients visited the ED after a fall or slip, 34.5% patients after a collision, and 15.2% patients after vehicle accidents.

**2.2 Model performance**

Figure 2 shows the ROC curve for all outcomes of mTBI sub-group in time-split validation set. The primary outcome (ICH) was 0.927 for AUROC on the time-split validation set. For the secondary outcomes, other TBIs than ICH obtained AUROC values of 0.862, operation obtained AUROC values of 0.828, and visit disposition weighted AUROC values of 0.932 respectively.

**Figure2. Receiver operating characteristic (ROC) curve for all outcomes on the time-validation set**

**
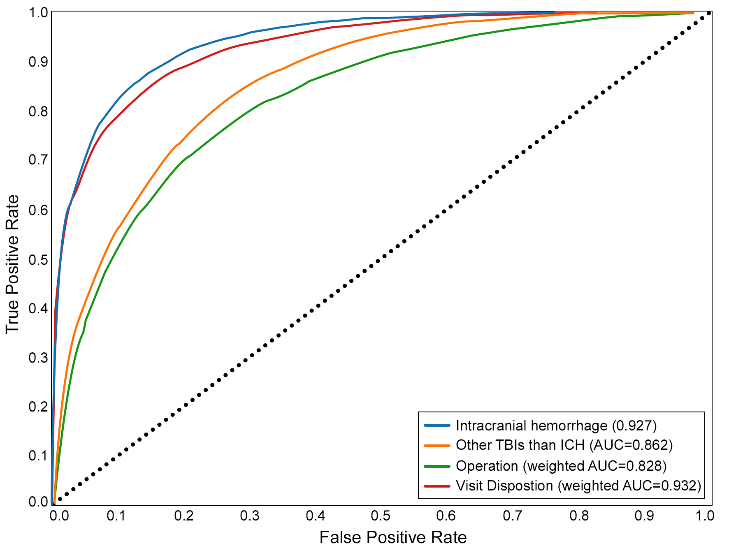
**
